# 2018: a year in review for *Open Biology*

**DOI:** 10.1098/rsob.190015

**Published:** 2019-01-30

**Authors:** David M. Glover

**Affiliations:** University of Cambridge, UK

A new year is with us again and *Open Biology* moves forward to meet new challenges in the ever-changing world of scientific publishing. Hopefully, we manage to keep the best of the old while keeping abreast of the new.

We are still managing to turn papers around with some speed. In 2018, the average time from submission to first decision was 30 days, and then it took an average of 25 days to get from the final decision to online publication. Why use a preprint server? Although we are equally happy if you do and are happy to sign articles from the popular servers, we hope that all of this makes our authors happy. Our readers are! In 2018, *Open Biology* articles received their highest usage to date with over 1.1 million downloads.

In the past few years, we have encouraged the submission of review articles from leaders in their fields. The response has been tremendous, and our review articles have become one of the hallmarks of the journal. We particularly encourage review articles that generate innovative and testable ideas and constructive discussions and/or critiques of their fields. In 2018, 62 invited reviews were submitted and 48 have already been published—almost double the 25 from 2017. The rest will follow shortly and be enhanced by the articles that are in the pipeline. We see this as an important role for the journal in providing a forum that stimulates the circulation and discussion of up-to-date results in developing fields in the widest possible way.

The journal is partnered with Altmetric which tracks the number of times articles are mentioned in the news, on blogs and in social media. Each *Open Biology* article features an ‘Altmetric donut’ in the Details tab. Some of the highest Altmetric scores include a perspective article on predicting virus emergence [1], a research paper on the role of a ‘molecular switch’ in Parkinson's Disease [2], a review on epichromatin and chromomeres [3] and developments in cancer immunotherapy [4].

It is not only our review articles that are catching attention. Notably, two articles also on rapamycin in fission yeast from the groups of Mitsuhiro Yanagida [5] and Janni Petersen [6] have stimulated lots of interest and led to additional articles on the subject. Two papers exploring unusual links between cancer and placental development on the one hand [7] and malaria on the other [8], have also caught a lot of attention.

There are some new developments in the journal in the past year that I must report. One of these is to compile papers into special interest group collections. One dear to my own heart is a Special Collection on Centrosome Biology. We have an open call for papers for this special collection of articles focused on the biology of the centrosome. By pulling our papers together, not only in monthly issues but also in topic groups, we aim to highlight exciting work in particular fields and in this example, improve knowledge on centrosome function, evolution and abnormalities. We aim to publish the centrosome full collection later in the Summer 2019.

In a similar vein, in February 2019, we will publish a mini collection of reviews in collaboration with neuroscientists based at the National Academy of Taiwan, Academia Sinica. The Academy celebrated its 90th anniversary in 2018, and this is also the 10th anniversary of their Neuroscience Program. We are delighted to put together this collection of articles to commemorate these anniversaries.

We are actively exploring numerous ways to distribute data and methods. *Open Biology* has partnered with BioStudies, a database of the European Bioinformatics Institute (EMBL-EBI) that collects all the data about a study in one place. Since the pilot in September 2017, 20% of authors have made their data accessible through a data package. It is in the year 2018 we introduced a reproducibility checklist for authors to complete during the submission process which will help to encourage transparency and reproducibility of data and experiments. The form is available alongside the manuscript.

We also have a new author feedback tool from Publons. This is a new feature which allows out authors to rate the quality of the feedback that they receive from reviews and also give them the opportunity to recognize those peer reviewers who have made an exceptional effort to improve the quality of their work. We recommend researchers to sign up to Publons keep a validated track-record of contributions across all journals they contribute to.

Royal Society Publishing is now on WeChat and we encourage any academics based in China to follow us and engage with our content using our QR code. Our marketing team will regularly post videos, articles and news from the whole of our portfolio of journals in order to reach as wide an audience as possible. If you use WeChat, please do let us know what you think about our account.

The Royal Society regards publishing as one of its important objectives in order to make our findings as scientists as widely available as possible. We ask for *your* support to *Open Biology* and the other journals of the society to reach this objective. I am grateful for so much support; from my fellow editors Christine Holt, Marek Mlodzik and Peter Parham; from our hard-working international editorial board; and for all the referees who have helped us in the past year. Above all my thanks go to Buchi Okereafor at Royal Society Publishing for her constant attention to the journal and to me and to my review article secretary, Kseniya Tyshkevych, for her endless patience and her tireless contributions.

We hope to see your article submitted to *Open Biology* in 2019 and wish you and all our readers a prosperous new year with lots of exciting new discoveries.
